# Retraction: ‘Investigating the bioavailability of graphene quantum dots in lung tissues via Fourier transform infrared spectroscopy’ by Tabish *et al*.

**DOI:** 10.1098/rsfs.2025.0083

**Published:** 2025-11-26

**Authors:** Interface Focus Editorial Office

**Affiliations:** ^1^ The Royal Society, London, UK

**Keywords:** graphene quantum dots, bioavailability, lung tissues

*Interface Focus.* **8**, 20170054 (Published online 20 April 2018). (doi:10.1098/rsfs.2017.0054).

The Editor-in-Chief in agreement with the Publisher has retracted this article. Following an investigation, we have found that figure 1 in the published article contains duplications. When the authors provided the raw data and a new version of figure 1, it was found that the images used to create the new version of figure 1 did not come from the raw data. Therefore, the data reported in figure 1 of this article are unreliable, and as a result, the Editor-in-Chief no longer has confidence in the article and so will issue a retraction according to our publishing policies of conduct.

Prof. Russell Foster FRS.

